# Low expression of Rap1GAP is associated with epithelial-mesenchymal transition (EMT) and poor prognosis in gastric cancer

**DOI:** 10.18632/oncotarget.14074

**Published:** 2016-12-21

**Authors:** Ya Yang, Jia Zhang, Yan Yan, Hui Cai, Min Li, Kai Sun, Jizhao Wang, Xu Liu, Jiansheng Wang, Xiaoyi Duan

**Affiliations:** ^1^ The Second Department of Thoracic Surgery, The First Affiliated Hospital of Xi’an Jiaotong University, Xi’an 710061, Shaanxi, China; ^2^ Department of Vascular Surgery, The First Affiliated Hospital of Xi’an Jiaotong University, Xi’an 710061, Shaanxi, China; ^3^ Department of Radiology, The First Affiliated Hospital of Xi’an Jiaotong University, Xi’an 710061, Shaanxi, China; ^4^ Department III of Radiation Oncology, The First Affiliated Hospital of Zhengzhou University, Zhengzhou 450000, Henan, China

**Keywords:** gastric cancer, RAP1 GTPase activating protein (Rap1GAP), E-cadherin, Matrix metalloproteinase-2, prognosis

## Abstract

Rap1GAP is a crucial tumor suppressor, but its role in gastric cancer (GC) is little investigated. In this study, we found that the expression of Rap1GAP was decreased in GC. Low expression of Rap1GAP was positively correlated with advanced pTNM stage, Borrmann types, tumor diameter and poor prognosis in patients with GC. Low expression of Rap1GAP correlated with loss of E-cadherin expression, and anomalous positivity of MMP2 expression. Multivariate analysis showed that low expression of Rap1GAP was an independent prognostic factor. Ectopic expression of Rap1GAP impaired cell migration and invasion, promoted the expression of E-cadherin and decreased the expression of MMP2. These results suggest that Rap1GAP functions as a novel suppressor of EMT and tumor metastasis in GC, and loss of Rap1GAP predicts poor prognosis in GC.

## INTRODUCTION

Rap1GAP, a 663-amino-acid protein with a molecular weight of 73 kDa, is the first identified member of the family of GTPase-activating proteins (GAPs) [1, 2]. Rap1GAP is a critical tumor suppressor gene that is downregulated in multiple cancers such as prostate cancer [3], breast cancer [4], oropharyngeal squamous cell carcinoma [5], pancreatic cancer [6], thyroid carcinoma [7], melanoma [8], colorectal cancer [9], B-cell lymphomas [10], kidney cancer [11], and acute myeloid leukemia [12]. Firstly, overexpression of Rap1GAP was found to inhibit the proliferation of rat thyroid cells [13]. Thereafter, overexpression of Rap1GAP in cancer cells impairs cell migration and invasion *in vitro* [5, 6, 9, 13–15], and inhibits tumor formation and metastasis *in vivo* [6, 14, 16, 17]. Rap1GAP also impairs cell-matrix adhesion in the absence of effects on cell-cell adhesion [15].

The molecular mechanism of Rap1GAP down-regulation in cancers is poorly understood. In human colon carcinoma cells, downregulation of Rap1GAP promotes tumor progression by altering cell/matrix and cell/cell adhesion [9]. In thyroid cells, overexpression of Rap1GAP impairs TSH/cAMP-stimulated p70S6 kinase activity and cell proliferation [13]. Rap1GAP impairs cell-matrix adhesion in the absence of effects on cell-cell adhesion [15]. In breast cancer, genomic mutation of Rap1GAP plays an important role in cancer progression [18]. However, in head and neck squamous cell carcinoma, Rap1GAP promotes invasion via induction of MMP9 secretion [19]. In myeloid leukemia cell lines, Rap1GAP promotes leukemia cell differentiation and apoptosis, but increases leukemia cell invasion *in vitro* by the upregulation of MMP9 [12]. To date, no study has reported Rap1GAP expression in GC patients. Therefore, in the present study, the expression of Rap1GAP in GC and its association with clinicopathological parameters and prognosis were investigated.

Epithelial–mesenchymal transition (EMT) is a complex biological program in which epithelial cells undergo a dramatic morphological change and switch to form mesenchymal cells. This switch occurs along with a reduction of epithelial marker proteins, such as E-cadherin, and an increase of mesenchymal markers, such as N-cadherin [20–22]. Substantial evidence suggests that EMT plays a critical role during embryonic development and cancer progression, the latter being involved in tumor invasion, metastasis, apoptosis and senescence resistance [23–25]. EMT is not only deemed a player in the initiation of the invasion and metastasis and but also a sign of powerful capacity to invade and metastasize [23, 26, 27]. Hence, the expression of E-cadherin and MMP2 were evaluated in GC, and the association of Rap1GAP with E-cadherin and MMP2 was also investigated. Furthermore, we explored the effect of Rap1GAP *in vitro*, and the corresponding changes in E-cadherin and MMP2 expression, and in migration and invasion capacity of GC cells.

## RESULTS

### Expression of Rap1GAP, E-cadherin and MMP2 in GC

**Figure 1:**
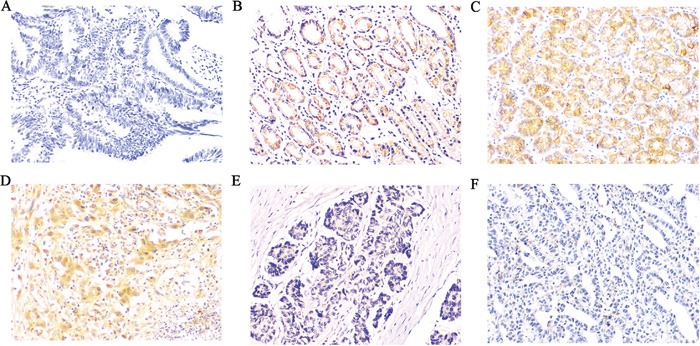
Immunohistochemical expression of Rap1GAP, E-cadherin and MMP2 in GC and para-carcinoma tissues **A**. Slides without primary antibody served as the negative control in GC tissues, **B, E**. Typical immunohistologic features with Rap1GAP expression in para-carcinoma and GC tissues, the Rap1GAP staining localized predominantly in the cytoplasm. **C, F**. Typical immunohistologic features with E-cadherin expression in para-carcinoma and GC tissues. **D**. Immunostaining for MMP2 was performed in GC; Magnifications, ×200.

The expression of Rap1GAP was determined in formalin-fixed, paraffin-embedded cancer tissues and para-carcinoma tissues by immunohistochemistry. Figure 1A as a negative control, as predicted, Rap1GAP was highly expressed in the normal follicular epithelial cells and was observed primarily in the cytoplasm (Figure 1B); however, Rap1GAP was not expressed in the stromal cells, endothelial cells and lymphocytes. Although Rap1GAP was detected in the tumor cells, there was a marked decrease in the staining intensity in these cells compared to the para-carcinoma tissues. Most of the GC samples were graded as negative (117 of 178, 65.73%), and 34.27% were positive (Table 1). These results are summarized in Figure 1E and confirmed a significant downregulation of Rap1GAP levels in GC.

**Table 1:** Differences in Rap1GAP, E-cadherin and MMP2 between the cancer tissues and para-carcinoma tissues

Group	Rap1GAP		P	E-cadherin		P	MMP2		P
	+	−		+	−		+	−	
cancer	61	117		70	108		132	46	
para-carcinoma	133	45	<0.001	148	30	<0.001	65	113	<0.001
total	194	162		218	138		197	159	

To investigate whether the loss of Rap1GAP expression in GC accounts for its EMT-like features and metastasis, we further analyzed the correlations between Rap1GAP and E-cadherin and MMP2. In GC, the epithelial protein loss frequency was 60.67% (108/178) for E-cadherin (Table 1 and Figure 1C, 1F), and aberrant metastasis protein expression frequencies were 74.15% (132/178) for MMP2 (Table 1 and Figure 1D), which was detected primarily in the cytoplasm. In addition, the expression levels of Rap1GAP and E-cadherin protein in GC cells were notably increased compared to 293T cells. Conversely, MMP2 was decreased in GC cells (Figure 3).

**Figure 2:**
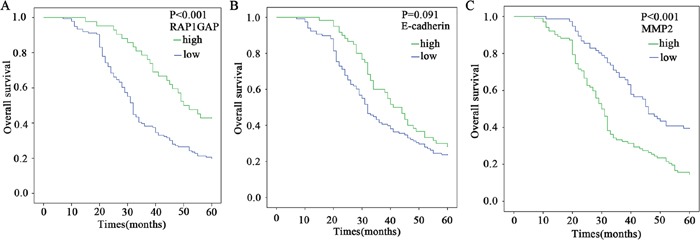
Kaplan-Meier survival analysis of Rap1GAP, E-cadherin and MMP2 expression levels The cumulative overall survival differences between the patients with high and low levels of protein expression. The P value was obtained using the log-rank test of the difference. **A**. Rap1GAP; **B**. E-cadherin; **C**. MMP2.

### Correlation between Rap1GAP expression with clinicopathological characteristics and E-cadherin and MMP2 expression in GC

The correlation between the Rap1GAP expression levels and the clinicopathological characteristics in GC was further analyzed, the results are summarized in Table 2. Low-expression of Rap1GAP was closely correlated with the pTNM stage, nodal involvement, metastasis, Borrmann types and tumor diameter, but not to the patients’ age, gender, the depth of invasion and histology. Moreover, the pTNM stage, depth of invasion, nodal involvement and histology were significantly correlated with a low expression of E-cadherin. High level of MMP2 was significantly correlated with the pTNM stage, depth of invasion, nodal involvement and metastasis (Table 2). Additionally, low expression of Rap1GAP was positively correlated with E-cadherin (r =0.433, P<0.001) and negatively associated with MMP2 expression (r= −0.403, P <0.001) (Table 3).

**Table 2:** Correlations between the clinicopathologic variables with Rap1GAP, E-cadherin and MMP2

Variables	N	Rap1GAP		P	E-cadherin		P	MMP2		P
		high	low		high	low		high	low	
Gender										
male	125	26	99		41	84		72	53	
female	53	16	37	0.177	19	34	0.694	30	23	0.902
Age										
≤60	94	26	68		33	61		51	43	
>60	84	16	68	0.177	27	57	0.676	51	33	0.384
TNM										
I	21	8	13		16	5		6	15	
II	34	12	22		19	15		14	20	
III	83	6	77	<0.001	13	70	<0.001	55	28	0.002
IV	40	16	24		12	28		27	13	
Depth of invasion										
T1	16	6	10		11	5		3	13	
T2	18	5	13		13	5		6	12	
T3	67	18	49	0.239	20	47	<0.001	39	28	<0.001
T4	77	13	64		16	61		54	23	
Nodal involvement										
N0	49	18	31		33	16		18	31	
N1	36	4	32		8	28		24	12	
N2	35	4	31	0.011	6	29	<0.001	21	14	0.007
N3	58	16	42		13	45		39	19	
Metastasis										
M0	138	26	112		49	89		73	65	
M1	40	16	24	0.006	11	29	0.346	29	11	0.027
Borrmann type										
I	22	10	12		9	13		11	11	
II	55	8	47		23	32		30	25	
III	62	14	48	0.037	20	42	0.156	38	24	0.778
IV	39	10	29		8	31		23	16	
Differentiation										
high	12	4	8		8	4		6	6	
moderate	57	15	42	0.538	26	31	0.001	28	29	0.226
poor	109	23	86		26	83		68	41	
Tumor diameter										
<3 cm	44	16	28		21	23		20	24	
3 cm-5 cm	54	7	47	0.025	18	36	0.053	33	21	0.187
>5 cm	80	19	61		21	59		49	31	

**Table 3:** Association between Rap1GAP with E-cadherin and MMP2

	Rap1GAP		r	P
	high	low		
E-cadherin				
high	30	30	0.433	<0.001
low	12	106		
MMP2				
high	9	93	−0.403	<0.001
low	33	43		

### Survival analysis

Survival curves were calculated using the Kaplan-Meier method and compared using the log-rank test. The patients with low Rap1GAP expression showed a more unfavorable prognosis than those with high expression (P<0.001) (Figure 2A and Table 4). The high levels of MMP2 expression had a statistically significant correlation with poor overall survival (P<0.001) (Figure 2C and Table 4); however, the low expression of E-cadherin was associated with no significant difference in the outcomes (P=0.091) (Figure 2B and Table 4). Moreover, as seen in Table 5, multivariate Cox analysis showed that a low expression level of Rap1GAP was an independent prognostic factor for patients with GC (P< 0.001)

**Figure 3:**
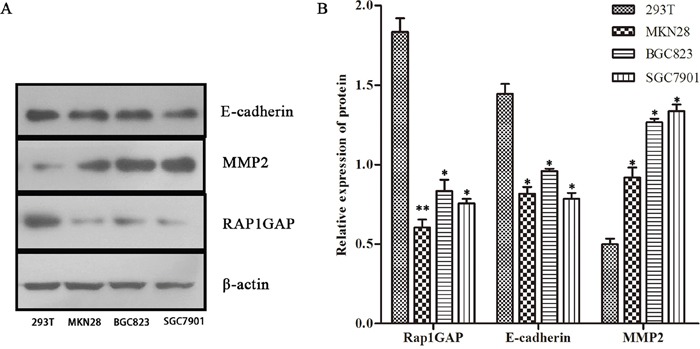
The protein expression of Rap1GAP, E-cadherin and MMP2 Rap1GAP and E-cadherin protein in GC cells were notably lower expression compared to 293T cells by western blotting. Conversely, MMP2 was upregulated in GC cells (*P < 0.05 and **P < 0.01)

**Table 4:** Univariate Analysis for Overall Survival

Variables	N	Overall survival		P
		Median±SE	95%CI	
Rap1GAP				
high	42	50.00±4.32	41.53-58.46	
low	136	32.00±0.89	30.37-33.62	<0.001
E-cadherin				
high	60	41.00±3.32	34.49-47.50	
low	118	32.00±1.80	28.45-35.54	0.091
MMP2				
high	102	30.00±1.12	27.80-32.19	
low	76	46.00±2.72	40.66-51.33	<0.001
Gender				
male	125	34.00±2.97	28.16-39.83	
female	53	38.00±3.63	30.86-45.13	0.973
Age				
≤60	94	40.00±3.87	32.40-47.59	
>60	84	32.00±1.13	29.77-34.22	0.223
TNM				
I	21	49.00±3.21	42.69-66.71	
II	34	45.00±5.83	33.57-56.42	
III	83	34.00±2.27	29.53-38.46	
IV	40	29.00±1.57	25.91-32.08	<0.001
Depth of invasion				
T1	16	46.00±8.66	29.01-62.98	
T2	18	52.00±15.91	20.81-83.18	
T3	67	35.00±4.60	25.98-44.01	
T4	77	31.00±1.45	28.14-33.86	0.005
Nodal involvement				
N0	49	55.00±9.79	35.79-74.20	
N1	36	43.00±7.80	27.22-58.78	
N2	35	36.00±3.54	29.04-42.95	
N3	58	29.00±2.53	24.03-33.96	<0.001
Metastasis				
M0	138	40.00±3.91	32.32-47.67	
M1	40	29.00±1.57	25.91-32.08	<0.001
Borrmann type				
I	22	39.00±7.62	24.06-53.93	
II	55	40.00±3.69	32.76-47.23	
III	62	34.00±3.50	27.14-40.85	
IV	39	30.00±1.77	27.51-34.48	0.502
Differentiation				
high	12	40.00±6.06	28.11-51.88	
moderate	57	39.00±6.11	26.97-51.02	
poor	109	32.00±1.74	28.59-35.41	0.107
Tumor diameter				
<3 cm	44	46.00±5.20	35.79-56.20	
3 cm-5 cm	54	34.00±1.83	30.40-37.51	
>5 cm	80	32.00±0.98	30.06-33.93	0.019

**Table 5:** Multivariate Cox Proportional Hazards Analysis for Overall Survival

Variables	Overall survival		P
	RR	95%CI	
Nodal involvement	1.249	1.042-1.498	0.016
Rap1GAP	2.578	1.531-4.341	<0.001
MMP2	1.502	1.032-2.188	0.034
Age	0.903	0.609-1.339	0.611
Gender	0.836	0.626-1.115	0.223
Depth of invasion	1.073	0.720-1.599	0.293
Metastasis	1.151	0.894-1.476	0.057
TNM	1.064	0.698-1.578	0.179
Borrmann type	0.844	0.637-1.129	0.259
Differentiation	0.930	0.674-1.285	0.622
Tumor diameter	0.877	0.466-1.691	0.717

### Overexpression Rap1GAP promoted the expression of E-cadherin and suppressed the expression of MMP2 in GC cells

**Figure 4:**
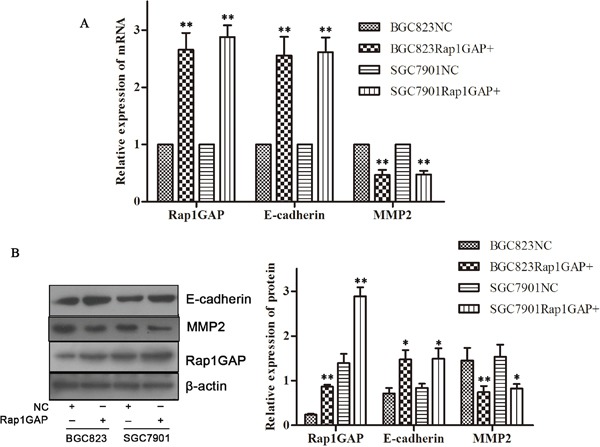
Overexpression Rap1GAP promoted the expression of E-cadherin and suppressed the expression of MMP2 in GC cells The GC cells were grown and transfected with the negative control (NC) or Rap1GAP CRISPR Activation Plasmid. Overexpression Rap1GAP improved the expression of E-cadherin (A, B) and, inversely, suppressed the expression of MMP2 (A, B) compared to NC group in mRNA level and protein level. (*P < 0.05 and **P < 0.01).

Rap1GAP was associated with expression of EMT features and aggressive phenotype of tumors. To illuminate Rap1GAP function in GC cells, BGC823 and SGC7901 cells are easy to metastasize distally and selected for the following experiments, we then transfected negative control (NC) and Rap1GAP CRISPR Activation Plasmid into GC cells. The expression of both E-cadherin mRNA and protein was increased compared to NC (P< 0.05; Figure 4); on the contrary, MMP2 was inhibited at the mRNA and protein level (P < 0.05; Figure 4). Rap1GAP promoted the expression of E-cadherin and suppressed the expression of MMP2 in GC cells. This suggested that E-cadherin and MMP2 might be the downstream genes of Rap1GAP.

### Overexpression Rap1GAP repressed the migration and invasion capacity of tumor cells

**Figure 5:**
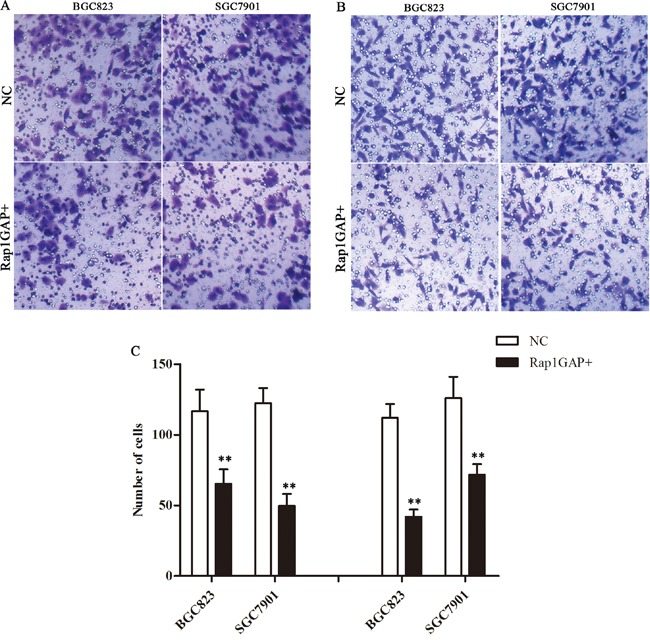
Overexpression Rap1GAP on the regulation of the migration and invasion capacity of tumor cells Overexpression Rap1GAP repressed the migration **A**. and invasion **B**. capacity of GC cells **C**. The graph shows the quantified data of the assay, migration (left) and invasion (right) (**P < 0.01).

Overexpression Rap1GAP cells were cultivated for 24 hours. Then, the migration assay and transwell invasion assay were used to detect the capacity of migration and invasion of Rap1GAP cells. As shown in Figure 5, both migration and invasive cell number were decreased following overexpression of Rap1GAP by Rap1GAP CRISPR Activation Plasmid (P< 0.05). Hence, Rap1GAP played an important role in the migration and invasion capacity of tumor cells.

## DISCUSSION

Rap1GAP is a Rap1 GTPase-activating protein that inhibits Rap1 activity by converting the active, GTP-bound from to the inactive, GDP-bound form [28]. Increasing evidences suggest that Rap1GAP function as a tumor suppressor through inhibiting proliferation, impairing cell invasion and metastasis and accelerating apoptosis [8, 14, 15, 17]; however, some studies indicated that an increased expression of Rap1GAP promoted tumor invasion and induction via MMP9 secretion [6, 12]. A critical question raised was whether Rap1GAP expression is clinically correlated with GC progression. In this study, we found that Rap1GAP expression in cancer tissues was lower than adjacent non-tumor tissues. Lower or lost expression of Rap1GAP has been reported in pancreatic cancer [6], thyroid tumors [7] and melanoma [8]. The low expression level of Rap1GAP was closely correlated with the pTNM stage, nodal involvement, metastasis, Borrmann types, tumor diameter and poor overall survival. Our findings of Rap1GAP low expression in both the GC samples and the cell lines indicate a role for Rap1GAP in gastric tumorigenesis.

The downregulation of Rap1GAP was influenced by various mechanisms, and Rap1GAP may exert cell type- and context-specific functions. In thyroid carcinoma cells, Rap1GAP expression was abolished by Ras transformation, promoter hypermethylation and loss of heterozygosity, and the downregulation of Rap1GAP promoted cell proliferation, migration and invasion [7, 14, 29]. In the head and neck squamous cell carcinoma (SCC), EZH2 repressed Rap1GAP by facilitating the trimethylationof H3K27 and hypermethylation of the Rap1GAP promoter, and the downregulation of Rap1GAP activated the activity of Rap1, and eventually promoted proliferation and invasion [30]. Rap1GAP has been shown to be frequently suppressed in malignant melanoma via promoter hypermethylation, which promoted melanoma cell proliferation, survival and migration [8]. The low expression levels of Rap1GAP in renal cell carcinoma cells was partly due to promoter hypermethylation, and attenuated the invasion of kidney cancer cells [31]. The downregulation of Rap1GAP was affected by various epigenetic mechanisms.

EMT along with a reduction of E-cadherin underlies the initiation of the invasion and metastasis of many human malignancies including GC [24, 32, 33]. One report showed that the overexpression of Rap1GAP decreases the expression of E-cadherin [31], however, our results showed that overexpression of Rap1GAP increased the expression of E-cadherin. Of particular relevance was a report that the E-cadherin protein levels were largely unchanged, but the accumulation of E-cadherin at cell/cell junctions reduced in the Rap1GAP-depleted cell lines. The alterations in cell/cell and cell/matrix adhesion are early steps in tumor metastasis supports a role for Rap1GAP depletion in tumor progression [9]. Rap1GAP expression was selectively suppressed in human thyroid carcinoma cell lines that neither unfolded an epithelial morphology nor expressed E-cadherin. The downregulation of Rap1GAP was correlated with the induction of a mesenchymal morphology and migratory behavior [1]. In another study, the overexpression of Rap1GAP did not enhance the dissociation of cell aggregates nor did it impair the accumulation E-cadherin at cell-cell contacts [15]. These data indicate that Rap1GAP is compatible with EMT and that the molecular mechanisms may be more complex than currently appreciated.

Some meta-analyses indicated that MMP2 expression may serve as an independent prognostic factor for GC [34–36]. Our results are similar to these former reports. Little is known about Rap1GAP and MMP2 in GC, and here we addressed a significant correlation between Rap1GAP and MMP2, simultaneously, our results showed that overexpression of Rap1GAP suppressed the expression of MMP2 and the migration and invasion capacity of tumor cells. Only have few studies have investigated the role of Rap1GAP in promoting the invasion of tumor cells via the upregulation of MMP9 secretion [7, 19]. Further investigations on the regulatory mechanisms of Rap1GAP and MMPs are needed.

In the present study, we found significant correlations between low Rap1GAP expression and the pTNM stage, nodal involvement, metastasis, Borrmann types, tumor diameter and poor overall survival. Rap1GAP is likely to serve as an important tumor suppressor in GC, and its loss during carcinogenesis contributes to tumor progression and metastasis. Rap1GAP was associated with expression of EMT features, aggressive phenotype of tumors and poor prognosis, suggesting Rap1GAP was a potential prognostic marker. Overexpression Rap1GAP promoted the expression of E-cadherin, suppressed the expression of MMP2 and repressed the migration and invasion capacity in GC cells. This suggested that Rap1GAP was one of therapeutic targets for GCs. But the regulation Rap1GAP with E-cadherin and MMP2 was intricate. Thus, further investigations are required.

## MATERIALS AND METHODS

### Ethics statement and patients

We collected tissues specimens of 178 gastric cancer patients from surgical cases in Department of Surgical Oncology, The First Affiliated Hospital, Xi’an Jiaotong University Medical School and Department of Surgical Oncology, the 215th Hospital of Shaanxi province between 2004 and 2009. The patients included 125 male and 53 female patients (ranging from 25 to 81 years of age). All of the patients were assessed according to the system for staging primary tumor/regional lymph nodes/distant metastasis (TNM) described in the AJCC Cancer Staging Manual. None of these 178 patients received neoadjuvant or adjuvant chemotherapy before the operation. The study was approved by the ethics committee and the human research review committee of Xi’an Jiaotong University.

### Immunohistochemical staining

The tissues specimens were fixed in neutral buffered formalin and embedded in paraffin wax. The sections of 4-mm thickness were cut and mounted on charged glass slides. Antigen retrieval was performed using citrate buffer at pH 6.0. The rabbit polyclonal antibody against Rap1GAP (Biosynthesis Bio-technology), MMP2 (Biosynthesis Bio-technology) and E-cadherin (CST) was performed. The streptavidin-peroxidase technique (Golden Bridge Int) was used. An irrelevant rabbit antiserum served as a negative control. The sections were stained with 0.02% diaminobenzidine (DAB) solution followed by counterstaining with hematoxylin.

### Evaluation of immunohistochemical analysis

The evaluation of Rap1GAP, E-cadherin and MMP2 expression was performed independently by two experienced pathologists who were blinded to the clinical data with consensus. The staining results were scored semi-quantitatively by calculating the immunostaining intensity and the percentage of positive malignant cells. The percentage of positive malignant cells was determined in at least 5 areas under 400 × magnifications and averaged. The mean percentage was scored as follows: 0 (0-5%); 1(6-25%); 2(26-50%); 3(51-75%), and 4 (76-100%). The staining intensities were scored as follows: no coloring, 0 point; slightly yellow, 1 point; brownish-yellow, 2 points; and tan, 3 points. Finally, the staining score was obtained by calculating the product of the staining intensity and the positive cell percentage, where ≤ 5 was defined as low expression and ≥ 6 as high expression.

### Cell lines and cell culture

The 293 T cell and human GC cell lines (MKN28, BGC823 and SGC7901) were obtained from the Cell Bank of Shanghai (Shanghai, China) and cultured in Dulbecco's Modified Eagle Medium (DMEM, Life Technologies)/high glucose medium supplemented with 10% heat-inactivated newborn calf serum at 37°C in a humidified incubator under a 5% carbon dioxide atmosphere. All of the experiments were performed with cells in the logarithmic phase of growth.

### Western blot analysis

Whole-cell lysates were prepared from the cell lines with a RIPA lysis buffer kit (Santa Cruz), and the protein concentrations were quantified using a Bio-Rad protein assay (Hercules). An equal amount of protein was separated using 10% and 12% SDS-PAGE and transferred to a polyvinylidene fluoride membrane (Millipore). The membranes were then probed with antibodies against the proteinsRap1GAP, E-cadherin and MMP2. The membranes were blocked in tris-buffered saline with tween containing 5% non-fat dry milk and then incubated overnight with the primary antibody, followed by incubation with the horseradish–peroxidase–conjugated antibodies at room temperature. The blots were developed using a peroxidase reaction and visualized with an ECL detection systems (Millipore). β-Actin was used as an internal positive control. The blots were analyzed densitometrically using the program Quantity One software (Hercules).

### Transfection

The GC cells were grown and transfected with the NC or Rap1GAP CRISPR Activation Plasmid (Santa Cruz) using Lipofectamine 2000, according to the manufacturer's protocol. After transfection for 48 h, the cells were harvested for RNA isolation and quantitative real-time PCR and western blot analysis of Rap1GAP expression.

### RNA isolation and quantitative real-time PCR

Total cellular RNA was isolated using a TRIzol reagent and reversely transcribed into cDNA using the Superscript II Reverse Transcriptase kit, according to the manufacturers’ instructions. The synthesized cDNA was then subjected to qPCR amplification using primers (Rap1GAP, 5′-GCA CTT TCT CGG CAA GGA GCA TTT-3′ and 5′-TGA CAT CAT GGT ATG TCC GGC ACT-3′; E-cadherin, 5′-AAG GAG GCG GAG AAG AGG AC-3′ and 5′-CGT CG TTA CGA GTC ACT TCA GG-3′; MMP2, 5′-CGC AGT GAC GGA AAG ATG TGG-3′ and 5′-AGA GCT CCT GAA TGC CCT TGA-3′; Glyceraldehydes-3-phosphate dehydrogenase (GAPDH), 5′-AGA AGG CTG GGG CTC ATT TG-3′ and 5′-AGG GGC CAT CCA CAG TCT TC-3′) under the following conditions: 95°C for 30 sec and 30 cycles of 95°C for 5 s, 60°C for 30 s, and 72°C for 60 sec for 30 cycles. Real-time PCR was performed in a multiplex PCR reaction with GAPDH as an internal control. Real-time PCR was performed with the Bio-Rad Thermocycler (iQ5) using SYBR green reagents (Hercules). The final products were verified by dissociation curves and data were analyzed by the relative quantification method normalized to GAPDH.

### Cell migration and invasion

Cell migration assay was performed using the Boyden chamber containing a membrane with an 8μm pore size (BD Bioscience). Cell invasion studies were also done using the Boyden chamber but membranes were pre-coated with Matrigel (1 mg/ml; BD Bioscience) matrices. In both cases, cells were seeded in wells at a density of 3–5 ×10^4^cells/200μl in DMEM medium containing 0.2% (v/v) FBS in the upper chamber. In the lower chamber, 600μl of DMEM media containing 10% FBS were added. After 24 h of incubation at 37°C in a 5% CO_2_ incubator, the chamber was removed, fixed, and stained with 1% crystal purple. Cells in the upper chamber were removed using a cotton swab. Cells migrating through the membrane and cells invading the matrix were randomly counted in five visual fields of each membrane at 200× magnification under a light microscope.

### Statistical analysis

Statistical analysis was performed using the SPSS software package (Version 16.0). A chi-square test was used to test the association of Rap1GAP, E-cadherin and MMP2 expression and the clinicopathological variables. The Spearman's rank correlation coefficient was used for analyzing the association of the E-cadherin and MMP2 expression levels with the Rap1GAP expression levels. Overall survival was defined as the time from the date of surgery to the date of the last follow-up or death from any cause. Survival curves were calculated using the Kaplan-Meier method and compared using the log-rank test. For multivariate analysis, the prognostic factors were analyzed using Cox's proportional hazard model. Student t test or one-way ANOVA was used to compare the normally distributed variables. The results were considered statistically significant if P < 0.05 (P< 0.05*, P < 0.01 **).

## Abbreviations

Rap1GAP: RAP1 GTPase activating protein
GC: gastric cancer
MMP2: Matrix metalloproteinase-2
EMT: Epithelial–mesenchymal transition
TNM: primary tumor/regional lymph nodes/distant metastasis
DAB: diaminobenzidine
DMEM: Dulbecco's Modified Eagle Medium
NC: negative control
